# Influence of Lithography Process Parameters on Continuous Surface Diffractive Optical Elements for Laser Beam Shaping

**DOI:** 10.3390/mi16050601

**Published:** 2025-05-21

**Authors:** Wenjing Liu, Axiu Cao, Junbo Liu, Hui Pang, Qiling Deng, Jian Wang, Song Hu

**Affiliations:** 1 National Key Laboratory of Optical Field Manipulation Science and Technology, Chinese Academy of Sciences, Chengdu 610209, China; 2 Institute of Optics and Electronics, Chinese Academy of Sciences, Chengdu 610209, China; 3 University of Chinese Academy of Sciences, Beijing 100049, China

**Keywords:** laser beam shaping, moving-mask lithography, diffractive optical element, continuous surface

## Abstract

To address the demand for laser beam-shaping techniques, we developed a one-step exposure process based on moving-mask lithography for the fabrication of a continuous-surface diffractive optical element (DOE) for laser beam shaping. The fabrication process is described in detail, and the influence of key parameters, such as pre-baking conditions, exposure gaps, development conditions, and post-baking conditions, of the lithography process on the microstructure profile of the DOE is analyzed. The reliability of the preparation method was verified through optical performance experiments. The speckle contrast, uniformity, and diffraction efficiency of the prepared linear beam-shaping element are 4.2%, 97.3%, and 87%.

## 1. Introduction

In recent years, rapid progress has been made concerning laser technology, and it has been widely applied in various fields, such as the medical, military, and information industries, among others. However, laser beams with a Gaussian energy distribution no longer meet some special requirements in practical applications. For example, laser-cutting technology requires a linear flat-top beam, exposure systems require a four-level flat-top beam, and laser projection systems require a rectangular uniform beam [1,2,3,4,5]. Therefore, it is critical to shape a Gaussian laser beam into a uniform beam of a specific shape.

The existing beam-shaping techniques are mainly divided into two categories: geometric and diffractive optics methods. The former is based on the principle of geometric optics and involves the use of refractive-type elements such as prisms, cylindrical lenses, and aspheric lenses to realize modulation [6,7,8]. The latter is based on using the principle of diffraction optics to realize the transformation of a beam. Compared with refractive elements, diffractive optical elements (DOEs) are smaller in terms of volume, lighter, and easier to integrate, and they allow arbitrary wavefront transformation, giving them wide application prospects in the field of beam shaping [9,10,11].

At present, most DOEs are multi-step structures, and the improvement in diffraction efficiency is governed by increases in the number of steps. Furthermore, multi-step DOEs need to be fabricated using multiple-alignment lithography and etching processes, for structures on the scale of hundreds of nanometers, presenting significant challenges in terms of fabrication complexity, technical difficulty, and production costs and making them unsuitable for mass production [12]. Several studies have described DOEs with continuous surfaces. In 1992, Prongue et al. proposed the design of a continuous-surface DOE using the two-step method. The resulting DOE had high diffraction efficiency and uniformity, but the design method converges slowly, which is not suitable for large DOEs [13]. In 2000, Arrizon et al. proposed an improved GS algorithm for designing a continuous-surface DOE, which also enabled high uniformity. However, the diffraction efficiency was low [14]. Based on the above research, our research group further improved the optimization algorithm to improve the diffraction efficiency, uniformity, and speckle contrast of the continuous-surface DOE [15]. However, the processing method for this continuous-surface DOE, an important subject, is little-studied. Laser direct writing, grayscale mask lithography, and moving-mask lithography can be used to prepare continuous-surface structures. Ni et al. proposed the fabrication of a large-scale high-numerical-aperture super-oscillatory lens by direct laser writing lithography. This technology can also be employed to fabricate continuous-surface DOEs [16]. However, its exposure system requires precise depth-of-focus adjustment to achieve the formation of continuous-surface profiles on photoresist, demanding exceptionally high precision in the fabrication process. This technology is not yet widely applicable for the fabrication of continuous-surface structures. Joao et al. prepared 2.5D Si master molds by direct write laser grayscale lithography [17]. Theoretically, this method could be applicable to the continuous-surface DOE structures proposed in this study, indicating potential fabrication prospects for future applications.

In the 1980s, Chen et al. proposed moving-mask lithography to fabricate elements with a continuous surface and a single-hole patterned mask [18]. In 2014, Nobuhiro et al. proposed the use of moving-mask lithography for fabricating 3D microstructure molding [19].

In recent years, our group has also conducted deeper research into this technology and successfully prepared continuous-surface micro-optical elements such as microlens arrays and spiral phase plates [20,21].

Based on the research noted above, this paper will focus on the fabrication of a continuous-surface DOE for laser beam shaping using a one-step exposure process based on moving-mask lithography. The influence of parameters in the key steps of the lithography process on the element is analyzed and discussed in detail. Ultimately, a linear beam-shaping element was prepared. This article is arranged as follows: Section 2 presents the structural parameters of the continuous-surface DOE and the fabrication method and process. Section 3 discusses the influence of processing parameters on the surface profile. Section 4 presents the results and details on the optical testing of the fabricated linear beam-shaping element. In the final section, we provide our conclusions.

## 2. Structural Parameters and Fabrication Methods

### 2.1. Structure Parameters of the Continuous-Surface DOE

Based on our previously proposed optimization algorithm [15], we designed a linear beam-shaping element with a continuous-surface profile. The designed wavelength (λ) is 632.8 nm, the diffraction angle is 4°, the pixel size (dx_1_) of the incident plane is 3 μm, the number of sampling points (M) is 512, and the focal length (F) of the Fourier lens is 350 mm. According to the Nyquist–Shannon sampling theorem, the sampling interval of the output plane satisfies the following equation: $dx_{2}=\lambda F/Mdx_{1}=144\;\mu m$. The number of pixel points (N) for the linear spot is 120, and the length of the linear spot satisfies the following equation: $dx_{2}\times N=17,280\;\mu m$. The phase depth (φ) obtained through simulation is 150.3 rad. According to Equation (1), the maximum etching depth (h) of the linear beam-shaping element is 33.13 μm on fused silica, where φ is the phase depth and n is the refractive index of fused silica, which is equal to 1.457.

(1) $$\varphi =\frac{2\pi h(n-1)}{\lambda }$$

**Figure 1:**
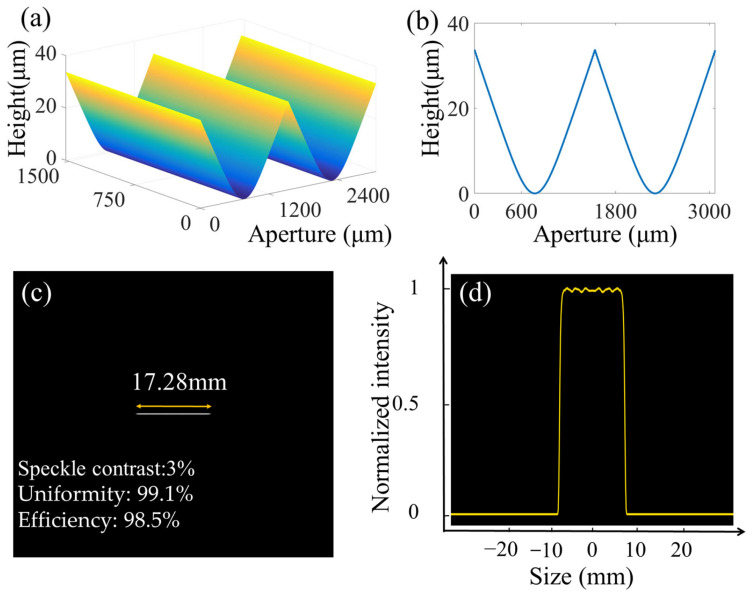
Design results regarding the continuous-surface DOE: (**a**) the distribution of the structure of the DOE, (**b**) the cross-section of the structure of the DOE, (**c**) the beam distribution, and (**d**) the cross-section of the intensity for linear beam shaping.

The design results regarding the continuous-surface DOE for linear beam shaping are shown in Figure 1. The period of the DOE is 1536 μm. Figure 1a displays the distribution of the DOE structure. Figure 1b shows the cross-section of the DOE structure: evidently, the profile is smooth, showing no abrupt changes. Figure 1c presents the reshaped beam, along with the linear distribution. Figure 1d shows the cross-section of the intensity. Evidently, the intensity distribution is very uniform, and the speckle contrast, uniformity, and diffraction efficiency were determined to be 3%, 99.1%, and 98.5%, respectively.

This design methodology offers high flexibility, enabling the transformation of Gaussian beams into customized two-dimensional distribution patterns (e.g., circular and rectangular). For generating rectangular spots with the same diffraction angle as given in this paper, two orthogonally aligned moving-mask exposures suffice during lithographic fabrication to produce the desired two-dimensional rectangular profiles.

### 2.2. Fabrication Method

**Figure 2:**
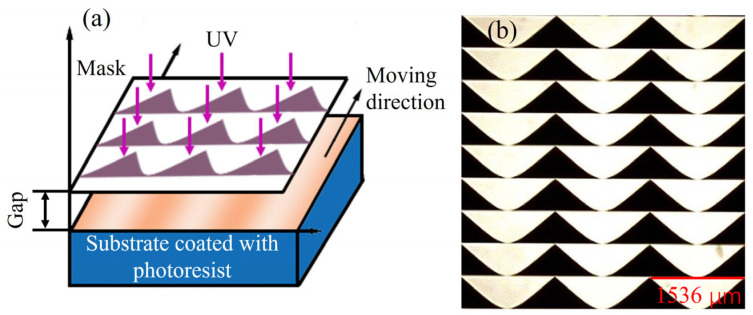
Moving-mask lithography: (**a**) principle and (**b**) mask.

We used a one-step exposure process based on moving-mask lithography to prepare the continuous-surface DOE. The principle is shown in Figure 2a. The substrate is pre-spin-coated with photoresist, which is irradiated with ultraviolet (UV) light. The mask is shown in Figure 2b, where the black area is the opaque area and the white area is the transmissive area. Notably, a certain gap is left between the substrate and mask to facilitate relative movement between the two. Such gaps also constitute an important parameter that affects the quality of the structural profile, which will be discussed later. During the exposure process, the mask moves continuously over a certain distance to adjust the exposure dose so as to form a continuous three-dimensional structure.

### 2.3. Preparation Process

**Figure 3:**
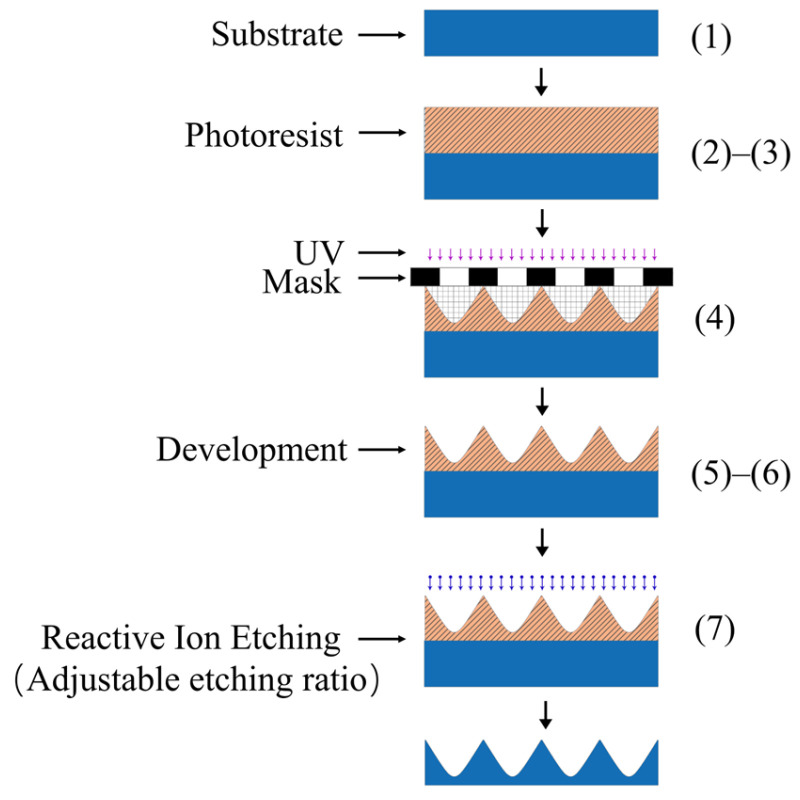
Process flow.

A continuous-surface DOE was fabricated through a combination of photolithography and reactive ion etching (RIE) methods. The main steps in the preparation process included (1) substrate cleaning, (2) photoresist coating, (3) pre-baking, (4) lithography, (5) development, (6) post-baking, and (7) etching. [20]. The process flow for these main steps is shown in Figure 3. The pattern on the photomask is transferred to the photoresist through exposure. Then, the structure of the photoresist is etched and transferred to a fused silica substrate through RIE. By adjusting the etching gas ratio (SF_6_ and CHF_3_) during RIE, the etching ratio between the photoresist and quartz substrate can be controlled precisely. In general, a maximum etching ratio of 1:2 can be achieved, indicating that the substrate material can achieve an etching depth twice that of the photoresist layer. Therefore, the depth of the structure on the photoresist can be appropriately reduced during photolithography, making profile accuracy easier to control.

In the preparation process, the surface profile of the DOE being developed on the photoresist is affected by many factors, such as the pre-baking, lithography, development, and post-baking parameters, which are analyzed and discussed in detail in the following sections.

## 3. Influence of Process Parameters

Fused silica with a thickness of 1 mm was selected as the optical substrate. The process of cleaning the substrates can be divided into four main steps. In the first step, acetone and alkaline cleaning agents were used to remove oils and organics from the substrate. In the second step, acidic cleaning agents and alcohol were used to soften the residue on the substrate. In the third step, deionized water was used to rinse the substrate. Finally, the substrate was dried using a hot plate.

Since the sag height of the designed structure is 33.13 μm, a thick-film photoresist (AZ50XT) was used to prepare the structure. In the actual processing phase, the thickness of the photoresist spin-coated through the determined uniform coating process was 26 μm. After exposure and development, the thickness of the photoresist was reduced to about 24 μm. Subsequently, the depth of the structure on the photoresist could be accurately measured, and the ratio of gas during RIE was set to achieve the target depth of 33.13 μm. The substrate, together with the photoresist, was spin-coated via a two-step spin-coating method using a spin coater (CHEMAT TECHNOLOGY SPIN-COATER KW-4A, Chemat Technology, Los Angeles, CA, USA). The substrate was first spin-coated for 4 s at a speed of 600 rpm and an acceleration of 600 rpm/s. Then, it was spin-coated again for 100 s at a speed of 400 rpm and an acceleration of 400 rpm/s. The thickness of the photoresist was determined to be 26 μm.

Lithography equipment (URE-2000/55YD) developed by the Institute of Optics and Electronics of the Chinese Academy of Sciences (Beijing, China) was used for exposure, with a wavelength of 365 nm. The exposure power was set to 3 mV, and the exposure time was set to 76.8 s. The Raybo developer (DM200-SE) was used for development. After completing the lithography process, we conducted surface profile measurements using a profilometer (Dektak XT, Bruker, Karlsruhe, Germany).

There are various nonlinear distortion factors that affect the quality of the surface profile of a thick-film photoresist. These parameters were analyzed using the control variable method.

### 3.1. Influence of Pre-Baking Conditions

Pre-baking is employed to evaporate the organic solvents in the photoresist in order to increase adhesion to the substrate. Excessively high temperatures and overly long baking times are not conducive to the volatilization of organic solvents in the photoresist and will reduce the adhesion of the photoresist to the substrate, impacting the sensitivity of the photoresist. If the temperature is too low and the baking time is too short, the organic solvents will not evaporate completely, and the photoresist will be too soft, leading to the contamination of the mask during exposure and the destruction of the microstructure on the photoresist [22].

In order to analyze the effect of pre-baking temperature and time, the effects of low-temperature, long-duration baking and high-temperature, short-duration baking were analyzed. The pre-baking temperature was set to 80 °C, 90 °C, and 100 °C, respectively, and the corresponding pre-baking time was set to 40 min, 20 min, and 10 min, respectively. Then, the photoresist was exposed using moving-mask lithography, and the exposure gap was set to 60 μm. A developing solution (AZ400K) was used for development. The ratio of the developing solution to deionized water was 3:7, and the development time was 150 s. The post-baking temperature and time for the developed substrate were set to 100 °C and 60 min, respectively.

**Figure 4:**
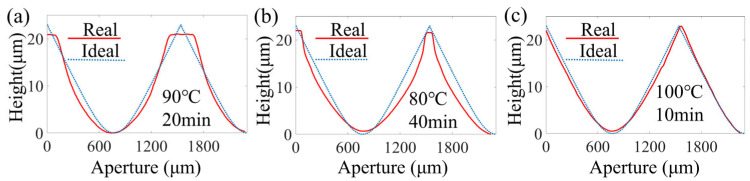
Fabrication under different pre-baking conditions: (**a**) a temperature of 80 °C and a duration of 40 min, (**b**) a temperature of 90 °C and a duration of 20 min, and (**c**) a temperature of 100 °C and a duration of 10 min.

The surface profiles of the photoresists fabricated under the three different pre-baking conditions were measured, as shown in Figure 4. The pre-baking conditions mainly affected the adjacent regions of the periodic structure. When the pre-baking temperature and time were 100 °C and 10 min, respectively, the surface profile was clearly closest to the ideal surface profile. Therefore, for this continuous-surface DOE, the high-temperature, short-duration pre-baking method was used in the subsequent steps.

### 3.2. The Effect of the Exposure Gap

**Figure 5:**
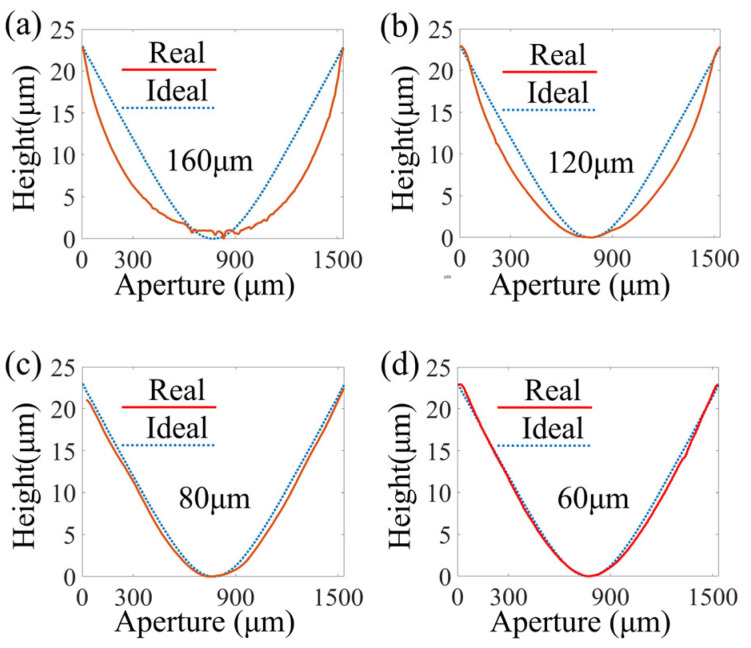
Fabrication with different exposure gaps: (**a**) 160 μm, (**b**) 120 μm, (**c**) 80 μm, and (**d**) 60 μm.

In the process of lithography, because of the creation of a certain gap between the substrate and mask, the effect of this gap on the profile of the DOE needs to be considered. According to the findings in Section 3.1, the high-temperature, short-duration pre-baking settings of 100 °C and 10 min were used to pre-bake the spin-coated photoresist. The exposure gaps were set to 160 μm, 120 μm, 80 μm, and 60 μm, and the subsequent development and post-baking conditions were the same as those used in Section 3.1. The surface profiles of the materials fabricated under the different exposure gaps were measured, as shown in Figure 5. Evidently, the exposure gap mainly affected the curvature of the surface profile. The greater the gap, the greater the distortion of the surface profile of the structure. When the gap was set to 60 μm, the actual surface profile was basically the same as the ideal surface profile. Therefore, the gap was set to 60 μm in the following experiments.

### 3.3. The Impact of Development Conditions

**Figure 6:**
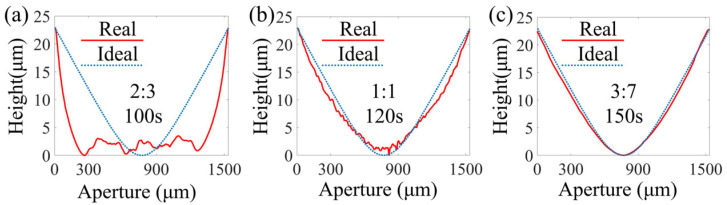
Fabrication with different developer concentrations and times: (**a**) a ratio of developer to deionized water of 2:3 and a development duration of 100 s, (**b**) a ratio of 1:1 and a development duration of 120 s, and (**c**) a ratio of 3:7 and a development duration of 150 s.

In development, a developer was used to develop the exposed pattern. Regarding the positive photoresist, the exposed part was dissolved by the alkaline developer, and the unexposed part was retained. The effects of developer concentration and development time on the surface profile were further examined. The pre-baking temperature and time were set to 100 °C and 10 min, respectively. The separation gap was set to 60 μm. The ratios of developer to deionized water were 2:3, 1:1, and 3:7, respectively, and the corresponding development times were 100 s, 120 s, and 150 s, respectively. The post-baking temperature and time were set to 100 °C and 60 min, respectively. The surface profiles of the materials fabricated under the different development conditions were measured, as shown in Figure 6. Evidently, when the ratio was 2:3 and the development time was 100 s, the distortion of the surface profile was maximum, with the bottom of the structure appearing uneven (Figure 6a). When the ratio was adjusted to 1:1 and the time was set to 120 s, the surface profile clearly improved compared with that in Figure 6a. However, the surface profile was still not smooth. When the ratio was adjusted to 3:7 and the time was set to 150 s, the surface structure was in a better state (Figure 6c). Therefore, for the development of a thick-film photoresist based on a continuous-surface DOE, it is necessary to reduce the concentration of the developer and increase the development time appropriately.

### 3.4. The Impact of Post-Baking Conditions

**Figure 7:**
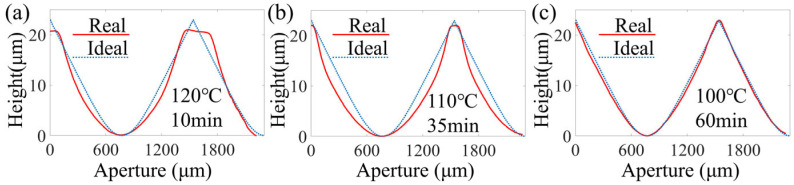
Fabrication using different post-baking conditions: (**a**) a temperature of 120 °C and a duration of 10 min, (**b**) a temperature of 110 °C and a duration of 35 min, and (**c**) a temperature of 100 °C and a duration of 60 min.

In order to improve the photoresist’s resistance to etching, post-baking after development had to be performed. The influence of different post-baking process parameters on the surface profile was analyzed. The pre-baking temperature and time were set to 100 °C and 10 min, respectively. The exposure gap, ratio of developer to deionized water, and development time were set to 60 μm, 3:7, and 150 s, respectively. The post-baking temperature was set to 120 °C, 110 °C, and 100 °C, and the corresponding time was set to 10 min, 35 min, and 60 min, respectively. The surface profiles measured at different post-baking temperatures and times are shown in Figure 7. When post-baked at 100 °C for 60 min, the corresponding surface profile of the photoresist clearly shows minimal distortion. Therefore, low-temperature, long-duration baking should be used in the post-baking stage.

## 4. Fabrication Results and Optical Testing

**Figure 8:**
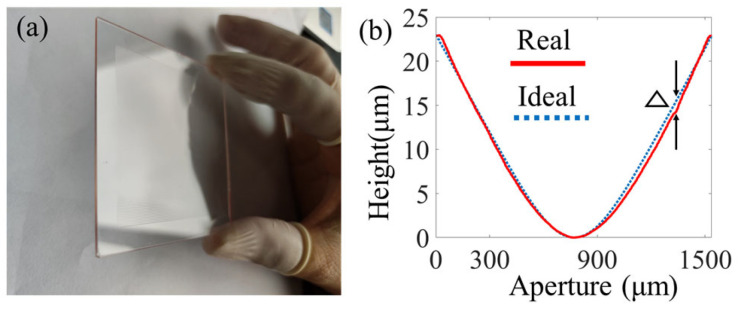
Fabrication results: (**a**) the linear beam-shaping element; (**b**) the surface profiles, where △ represents the maximum profile mismatch between the ideal and fabricated surface profiles.

At this stage, the linear beam-shaping element designed in Section 2.1 was fabricated. Fused silica with a thickness of 1 mm was selected as the optical substrate. Based on the discussion in Section 3, detailed process parameters were determined, as shown in Table 1. The continuous-surface DOE for linear beam shaping was prepared as shown in Figure 8a. The surface profile of the actual prepared structure measured using a step profilometer (stylus profiler system, Dektak XT, Broker, Karlsruhe, Germany) was compared with the ideal profile, revealing a maximum profile mismatch of 1.1 μm (Figure 8b). The root mean square error (RMS) was 3%, which is suitable for the surface shape requirements.

**Figure 9:**
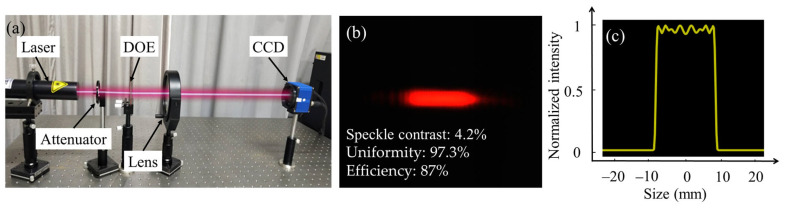
(**a**) Optical system, (**b**) test results, and (**c**) the cross section of the intensity of the linear spot.

Furthermore, to verify the performance of the linear beam-shaping element, a beam-shaping experiment was carried out. The optical system is shown in Figure 9. The laser was first modulated by the DOE and the lens. The focal length, F, of the lens was 350 mm, and the CCD (HR16000CTLGEC) was placed at the focus plane of the lens to record the spot formed. The length of the linear spot collected by the CCD was 1.7 cm, which is basically consistent with the simulation results, namely, 1.728 cm, given in Section 2.1, as shown in Figure 9b. The intensity distribution was relatively uniform across the cross-section of the linear spot, as shown in Figure 9c. The speckle contrast, uniformity, and diffraction efficiency were 4.2.%, 97.3%, and 87%. In the experiment, the width of the spot formed was determined by the width of the input spot, which was 0.36 mm. Therefore, the actual spot that formed was thicker, as shown in Figure 9b, exhibiting a width consistent with that of the input spot.

## 5. Conclusions and Summary

The continuous-surface DOE for beam shaping proposed in this paper demonstrates high efficiency, high uniformity, and low speckle. A one-step exposure process based on moving-mask lithography was employed for the fabrication of this continuous-surface DOE for laser beam shaping, constituting an efficient and inexpensive method. The influence of key parameters of the lithography process on the surface profile was analyzed in detail. Hopefully, this study can provide a reference for the future study of the preparation of continuous-surface DOEs, which are highly suitable for the batch production of beam-shaping devices with significant potential for practical applications. Linear beam-shaping elements with continuous surfaces, offering better performance, can be widely used in many fields, such as the medical, military, and information industries, among others.

## Figures and Tables

**Table 1:** Process parameters.

Process Flow	Process Parameters
Photoresist coating	First spin coating: 4 s, 600 rpm, and 600 rpm/s. Second spin coating: 100 s, 400 rpm, and 400 rpm/s.
Pre-baking	Temperature: 100 °C; time: 10 min
Lithography	Moving distance, 1536 μm; moving interval, 1 μm every 0.05 s; intensity, 3 mV; separation gap, 60 μm.
Development	Ratio of developer to deionized water, 3:7; time, 150 s.
Post-baking	Temperature: 100 °C; time: 60 min.

## Data Availability

Data will be provided on request due to privacy through the corresponding author (Axiu Cao) of this article.
